# Introducing nhj.nl—a website companion to the Netherlands Heart Journal

**DOI:** 10.1007/s12471-025-01998-5

**Published:** 2025-10-15

**Authors:** Pim van der Harst

**Affiliations:** Department of Cardiology, Division of Heart and Lungs, University Medical Centre Utrecht, Utrecht, The Netherlands

I am pleased to introduce **nhj.nl**, the Dutch-language companion site to the *Netherlands Heart Journal*. Its purpose is straightforward: to strengthen the journal’s position and offer readers additional relevant and accessible content—clearly, concisely, and in Dutch. This companion website will coexist with our fully open-access publications on SpringerLink: https://link.springer.com/journal/12471.

The site will be officially launched during the NVVC Congress on 6 November.

The website features Dutch-language summaries that link directly to the full articles on SpringerLink, news items about NHJ papers, cardiology challenges, including Rhythm and Image Puzzles and Heart Beats, extra online material for the new *Facts & Figures* section, additional information referred to from print, authors recommending their papers, short author videos, the option for readers to respond, NVVC abstracts and endorsements, and submitted letters from the field.

To make navigation effortless, nhj.nl also introduces clear content categories that bring related group items together. For example, you will find dedicated headings under which you can easily find all published NVVC scientific statements and endorsements in one place—without needing to search across multiple issues. In this way, the site complements SpringerLink by lowering the threshold for quick orientation and providing a direct path to the full scientific record.

I invite you to visit and bookmark nhj.nl, and to make active use of it by reading, responding to, and sharing materials that support clinical practice, continuing education, and scientific discussion.

